# A Pyrrole-Imidazole Polyamide Targeting Human USF1 Binding Suppresses High Glucose-Induced TGF-β1 and Osteopontin Expression in Human Renal Mesangial Cells

**DOI:** 10.3390/cells15191818

**Published:** 2026-10-06

**Authors:** Yurie Akiya, Hiroki Kobayashi, Akiko Tsunemi, Noboru Fukuda, Masanori Abe

**Affiliations:** 1 Division of Nephrology, Hypertension, and Endocrinology, Department of Internal Medicine, Nihon University School of Medicine, 30-1 Oyaguchi Kami-chou, Itabashi-ku, Tokyo 173-8610, Japan; 2 Division of Nephrology and Endocrinology, Ukima Chuo Hospital, Tokyo 115-0052, Japan

**Keywords:** diabetic nephropathy, upstream stimulatory factor 1, transforming growth factor-β1, osteopontin, pyrrole-imidazole polyamide

## Abstract

Transforming growth factor-β1 (TGF-β1) is a key profibrotic mediator in diabetic kidney disease. Upstream stimulatory factor 1 (USF1) enhances TGF-β1 transcription through an E-box/glucose-response element in the TGF-β1 promoter under high-glucose conditions. We designed a sequence-specific pyrrole-imidazole (PI) polyamide targeting the human USF1-binding site in the TGF-β1 promoter and evaluated its activity in human renal mesangial cells (HRMCs). A target PI polyamide and a mismatch PI polyamide were synthesized. DNA binding and inhibition of the USF1–DNA interaction were assessed by gel mobility shift assays. HRMCs were exposed to high glucose with or without PI polyamides, and TGF-β1 and osteopontin expression were quantified by real-time PCR and Western blotting. The target PI polyamide bound the intended promoter sequence and reduced recombinant USF1 binding in vitro. In high-glucose-exposed HRMCs, the target PI polyamide suppressed TGF-β1 mRNA and protein expression, whereas the mismatch PI polyamide did not suppress TGF-β1 expression. The target PI polyamide also reduced osteopontin mRNA expression. These findings provide proof-of-concept that PI polyamide-mediated targeting of the human USF1–TGF-β1 axis can attenuate high-glucose-associated profibrotic responses in HRMCs.

## 1. Introduction

Diabetic kidney disease remains a leading cause of chronic kidney disease and kidney failure worldwide. Progressive mesangial expansion, glomerulosclerosis, and tubulointerstitial fibrosis are key pathological features of this condition. Among the mediators implicated in these processes, transforming growth factor-β1 (TGF-β1) has been recognized as a central profibrotic cytokine that promotes extracellular matrix accumulation and structural kidney injury in diabetic kidney disease [1,2,3].

Pyrrole-imidazole (PI) polyamides are sequence-specific DNA-binding molecules that recognize double-stranded DNA in the minor groove according to defined pyrrole/imidazole pairing rules [4,5]. Because of their high binding affinity, nuclease resistance, and ability to enter cells and nuclei without a delivery system, PI polyamides have attracted attention as transcriptional regulators. Previous studies have shown that PI polyamides targeting the TGF-β1 promoter can suppress progressive renal injury in experimental models, including hypertensive nephropathy and diabetic nephropathy [6,7].

Unlike conventional approaches that inhibit TGF-β1 signaling after its production, PI polyamides can interfere with transcription by binding directly to predefined promoter sequences. This sequence-directed mechanism may be particularly relevant to glucose-responsive transcription, in which transcription factor occupancy is enhanced under high-glucose conditions. Importantly, because PI polyamides recognize specific nucleotide sequences, activity against a rodent promoter sequence cannot necessarily be extrapolated to the corresponding human promoter. Direct evaluation of sequence-specific binding and transcriptional suppression at the human TGF-β1 promoter is therefore an important step toward further translational development.

High-glucose conditions activate several signaling pathways and transcription factors that contribute to diabetic kidney injury [8]. In mesangial cells, upstream stimulatory factor 1 (USF1) binds to the glucose-response element/E-box motif within the human TGF-β1 promoter and enhances TGF-β1 transcription under high-glucose conditions [9,10]. Experimental studies have also supported a pathogenic role of USF1 in diabetic kidney disease, showing that genetic disruption of USF1 attenuates TGF-β1 upregulation, albuminuria, and mesangial matrix accumulation [9,11]. These findings suggest that the USF1–TGF-β1 axis is a rational therapeutic target in diabetic kidney disease.

However, although a USF1-targeting PI polyamide has shown beneficial effects in rodent diabetic nephropathy models [7], its activity against the human TGF-β1 promoter has not been sufficiently examined. Human renal mesangial cells are a relevant model for this question because high glucose stimulates autocrine TGF-β1 signaling in these cells [12], and osteopontin has been linked to albuminuria and glomerular injury [13]. Therefore, in the present study, we designed a PI polyamide targeting the human USF1-binding site in the TGF-β1 promoter and examined whether it suppresses high glucose-induced TGF-β1 and osteopontin expression in human renal mesangial cells.

## 2. Materials and Methods

### 2.1. Design and Synthesis of a PI Polyamide Targeting Human USF1 Binding on the TGF-β1 Promoter

**Figure 1:**
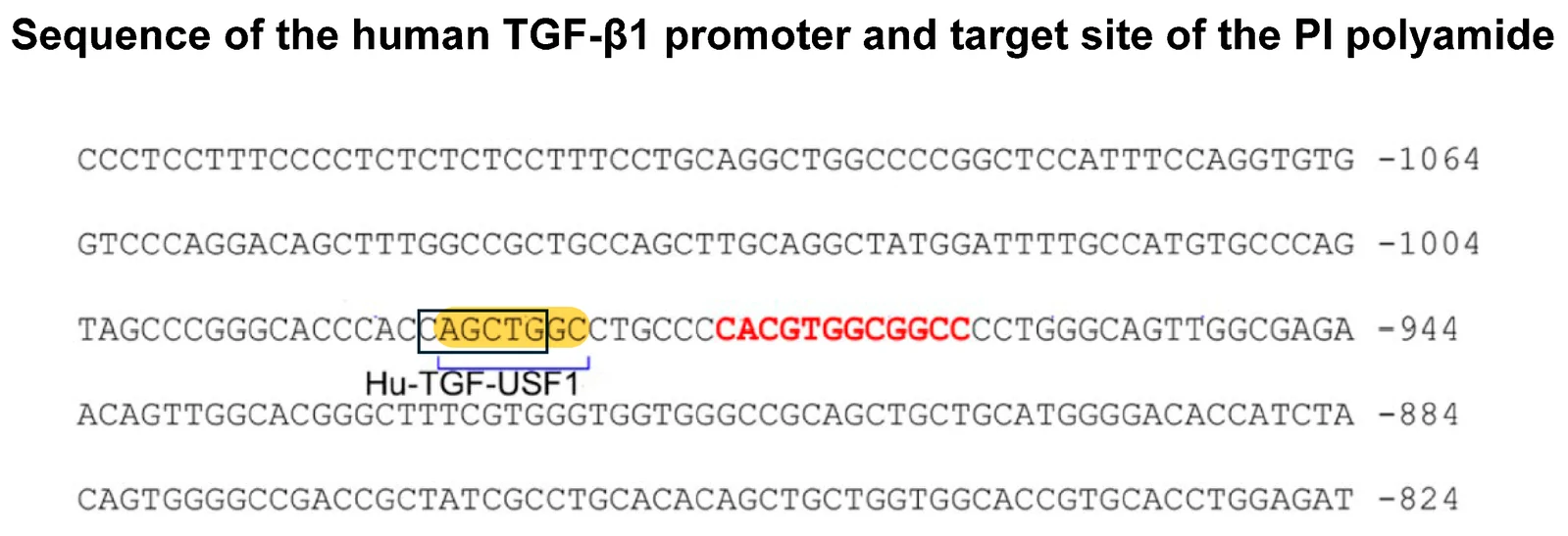
Sequence of the human transforming growth factor-β1 (TGF-β1) promoter and target site of the PI polyamide. The red sequence indicates the glucose-response element (GlRE). The PI polyamide was designed to span the E-box motif (CAGCTG/CANNTG motif) within the human TGF-β1 promoter. The area enclosed in a square indicates the E-box motif (CAGCTG/CANNTG motif), and the area marked in yellow indicates the binding site for PI polyamide.

The sequence of the human TGF-β1 promoter is shown in Figure 1. The red sequence indicates the glucose-response element (GlRE) in the human TGF-β1 promoter, and the area enclosed in a square indicates the E-box motif (CAGCTG/CANNTG motif). We designed a PI polyamide spanning the E-box motif (CAGCTG/CANNTG motif) sequence adjacent to the GlRE within the TGF-β1 promoter.

PI polyamides were synthesized by automatic solid-phase synthesis. Briefly, the resin was first washed with dimethylformamide (DMF), and the Fmoc group was removed with 20% piperidine/DMF. After washing with methanol, coupling was performed for 60 min with each monomer in the presence of 1-(bis(dimethylamino)methylene)-5-chloro-1H-benzotriazolium 3-oxide hexafluorophosphate and diisopropylethylamine (4 eq each). The resin was then washed with methanol, capped with acetic anhydride/pyridine, and washed again with DMF. After removal of the Fmoc group from the Fmoc-β-alanine-Wang resin, the resin was successively washed with methanol. The coupling step with Fmoc-amino acid, followed by methanol washing, was repeated until the full sequence had been synthesized. The N-terminal amino group was then protected, the resin was washed with DMF, and the reaction vessel was drained.

**Figure 2:**
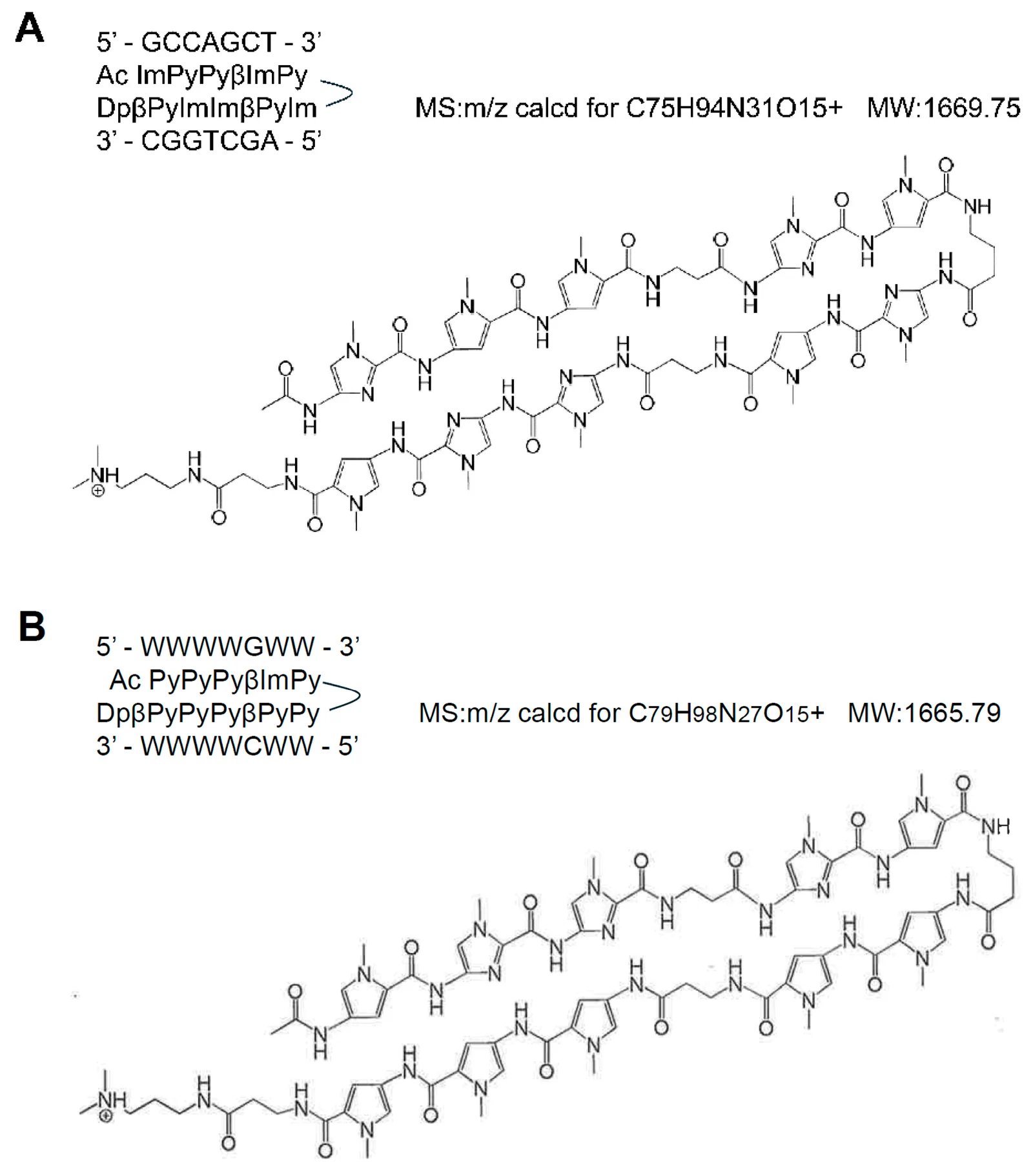
Structures of the pyrrole-imidazole (PI) polyamide targeting the human TGF-β1 promoter. The PI polyamides were synthesized by solid-phase methods and purified by high-performance liquid chromatography (HPLC) (0.1% AcOH/CH3CN, 0% to 50% linear gradient over 40 min; detection at 254 nm using a Chemcobond 5-ODS-H column). (**A**) Structure of the upstream stimulatory factor 1 (USF1) -targeting PI polyamide. (**B**) Structure of the mismatch PI polyamide.

For cleavage, 5 mL of 91% trifluoroacetic acid, 3% triisopropylsilane, 3% dimethyl sulfide, and 3% water per 0.1 mmol resin was added, and the synthetic polyamides were isolated by cold ethyl ether precipitation. A second cleavage step was then performed with 5 mL of N,N-dimethylaminopropylamine per 0.1 mmol resin at 50 °C overnight, and the synthetic polyamides were again isolated by cold ethyl ether precipitation. Finally, the polyamides were purified by high-performance liquid chromatography (HPLC) using a PU-980 HPLC pump, a UV-975 HPLC UV/VIS detector (Jasco, Easton, MD, USA), and a Chemcobond 5-ODS-H column (Chemco Scientific, Osaka, Japan). The structures of USF1- targeting PI polyamide and mismatch PI polyamide are shown in Figure 2.

### 2.2. Gel Mobility Shift Assays and DNA Binding Assays for USF1 PI Polyamide

To confirm binding of the designed USF1 PI polyamide to the target double-stranded DNA, gel mobility shift assays were performed. The following oligonucleotides were synthesized for the USF1 PI polyamide: sense, FITC-ACCCACCAGCTGGCCTGCCCC; antisense, GGGGCAGGCCAGCTGGTGGGT, and mutated DNA: sense, FITC-ACCCACCAGATGCCCCGCCCC; antisense, GGGGCGGGGCATCTGGTGGGT.

Oligonucleotides (10^−5^ M) were denatured at 95 °C for 5 min using a thermal cycler, cooled to allow annealing, and used as double-stranded DNA. The double-stranded DNA and PI polyamide (10 µM) were incubated in binding buffer containing 20% glycerol, 50 mM dithiothreitol, 5 mM MgCl_2_, 2.5 mM EDTA, 250 mM NaCl, and 50 mM Tris-HCl (pH 7.5) for 1 h. Samples were then electrophoresed on a 20% polyacrylamide gel for 1 h and visualized using a Fusion Solo System (Vilber Lourmat, Collégien, France).

To determine whether the USF1 PI polyamide inhibited the binding of USF1 to DNA, DNA binding assays were performed using the same double-stranded oligonucleotides and recombinant human USF1 protein (ag17789, Proteintech, Rosemont, IL, USA). Double-stranded DNA (10 µM) and USF1 PI polyamide (50 µM) were incubated in the same binding buffer for 1 h, followed by addition of recombinant human USF1 protein (1 µg). The samples were electrophoresed on a 20% polyacrylamide gel for 1 h and visualized using the Fusion Solo System.

### 2.3. Culture of Human Renal Mesangial Cells and Glucose Stimulation with USF1 PI Polyamide

Human renal mesangial cells (HRMCs; ScienCell Research Laboratories, Carlsbad, CA, USA; catalog no. 4200) were cultured in Dulbecco’s modified Eagle medium (DMEM; 08456-36, Nacalai Tesque, Kyoto, Japan) supplemented with 10% fetal bovine serum (FBS; CCP-FBS-BR-500, Cosmo Bio, Tokyo, Japan) and penicillin-streptomycin (09367-34, Nacalai Tesque, Kyoto, Japan) in a humidified atmosphere containing 5% CO_2_ at 37 °C. Cells were seeded in 6-well plates in normal-glucose DMEM (5.6 mM glucose) containing 10% FBS and grown to 80% confluence. The medium was then replaced with DMEM containing 0.5% FBS for 24 h for serum starvation.

Three hours before the end of serum starvation, the USF1 PI polyamide or mismatch PI polyamide was added to the culture medium at final concentrations of 10^−12^, 10^−11^, 10^−10^, and 10^−9^ M in normal-glucose DMEM (5.6 mM glucose; 08456-36, Nacalai Tesque, Kyoto, Japan). The mismatch PI polyamide was tested at the same final concentrations as the USF1 PI polyamide. For high-glucose stimulation, cells were subsequently cultured in high-glucose DMEM (25 mM glucose; 08458-16, Nacalai Tesque, Kyoto, Japan) containing the same concentrations of the respective PI polyamides for 24 h for mRNA analysis. For protein analysis, HRMCs were cultured with 10^−9^ M USF1 PI polyamide or mismatch PI polyamide in high-glucose DMEM for 48 h. Real-time PCR was performed to evaluate TGF-β1 and osteopontin mRNA expression, and Western blot analysis was performed to evaluate TGF-β1 protein expression.

### 2.4. RNA Extraction and Real-Time PCR Analysis

Total RNA was extracted from HRMCs using TRIzol reagent (15596018, Invitrogen, Carlsbad, CA, USA). cDNA was synthesized using ReverTra Ace qPCR RT Master Mix with gDNA Remover (FSQ-301, TOYOBO, Osaka, Japan).

To evaluate TGF-β1 mRNA expression, quantitative real-time PCR was performed using Premix Ex Taq (Probe qPCR), ROX plus (RR39LR, Takara, Shiga, Japan), and a StepOnePlus Real-Time PCR System (Applied Biosystems, Foster City, CA, USA). The amplification conditions were 95 °C for 20 s, followed by 40 cycles of 95 °C for 1 s and 60 °C for 20 s.

To evaluate osteopontin mRNA expression, quantitative real-time PCR was performed using KOD SYBR qPCR Mix (QKD-201, TOYOBO, Osaka, Japan) and the same PCR system. The amplification conditions were 98 °C for 2 min, followed by 40 cycles of 98 °C for 10 s, 60 °C for 10 s, and 68 °C for 30 s. mRNA expression levels were evaluated using the standard curve method. Based on the calibration curve data, each target mRNA level was normalized to 18S rRNA, and relative quantification was performed (Supplementary Table S1).

### 2.5. Western Blot Analysis

Cultured HRMCs were lysed with RIPA buffer (08714, Nacalai Tesque, Kyoto, Japan). Equal amounts of total protein were prepared with sample buffer (#161-0747, Bio-Rad Laboratories, Hercules, CA, USA), boiled at 95 °C for 5 min, separated on 10% polyacrylamide gels, and transferred to PVDF membranes using an EPS 601 electrophoresis power supply (GE Healthcare, Chicago, IL, USA). The membranes were blocked before incubation with primary antibodies.

The membranes were then incubated overnight at 4 °C with anti-TGF-β Rabbit monoclonal antibody (#3709, Cell Signaling Technology, Danvers, MA, USA; 1:1000) and rabbit anti-GAPDH polyclonal antibody (ab9485, Abcam, Cambridge, UK; 1:8000) in TBST solution (10 mM Tris-HCl, pH 8.0, 150 mM NaCl, and 0.05% Tween 20). The membranes were then incubated with horseradish peroxidase-conjugated goat anti-rabbit IgG (111-036-003, Jackson ImmunoResearch Laboratories, West Grove, PA, USA) for 1 h at room temperature and washed three times with TBST for 5 min each. Band intensities were quantified using ImageJ software (version 1.53k, National Institutes of Health, Bethesda, MD, USA) [14]. Data are presented relative to the Normal glucose group.

### 2.6. Statistical Analysis

Data are presented as means ± SE. Normality was assessed using the Shapiro–Wilk test. Outliers were evaluated using the Smirnov-Grubbs test for qPCR analysis, whereas no outlier testing was performed for Western blot analysis. Statistical analyses were performed using one-way analysis of variance followed by Dunnett’s multiple-comparison test in GraphPad Prism version 10.6.1 for Windows (GraphPad Software, Boston, MA, USA). Values of *n* represent biological replicates. A *p*-value < 0.05 was considered statistically significant.

## 3. Results

### 3.1. Gel Mobility Shift Assay for USF1 PI Polyamide

**Figure 3:**
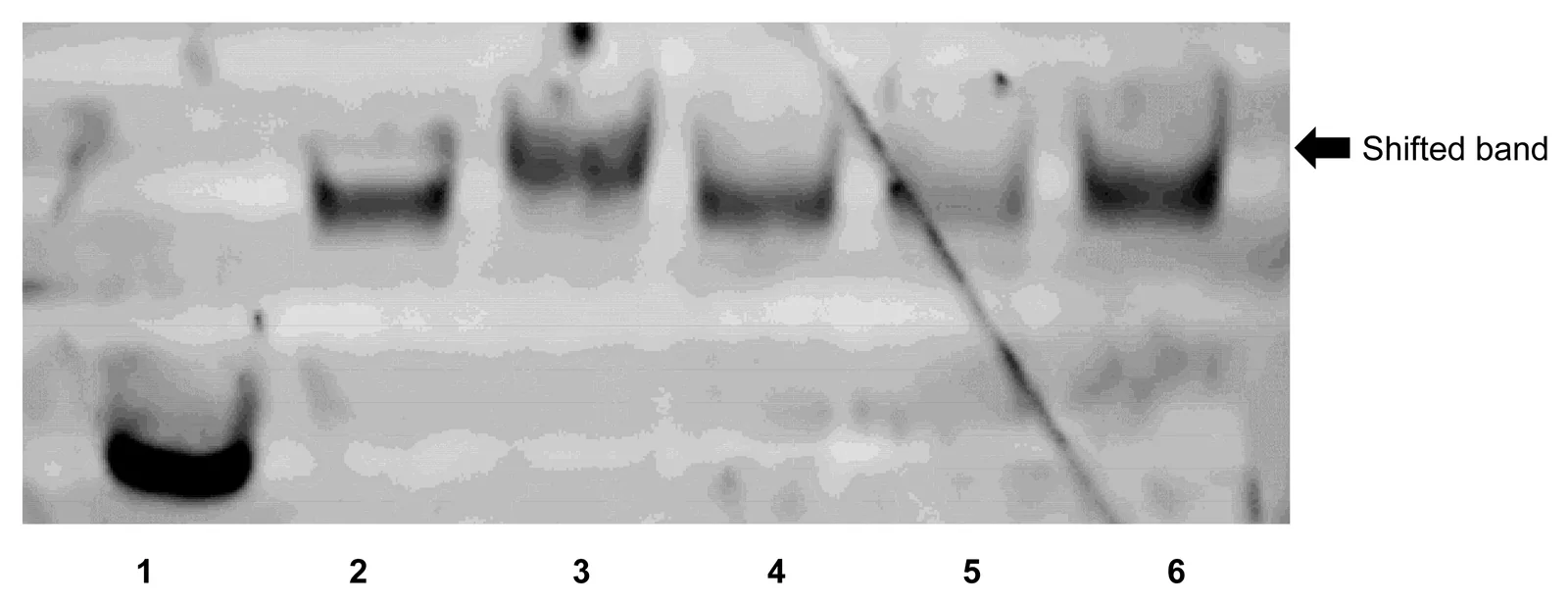
Gel mobility shift assay of the USF1-targeting pyrrole-imidazole (PI) polyamide. Sense and antisense oligonucleotides corresponding to the target sequence were synthesized, denatured at 95 °C, and cooled for annealing to generate double-stranded DNA. The double-stranded oligonucleotides were incubated with the PI polyamides in binding buffer and subjected to polyacrylamide gel electrophoresis. Lane 1, FITC-labeled single-stranded DNA; Lane 2, FITC-labeled double-stranded DNA (FITC-dsDNA); Lane 3, FITC-dsDNA + USF1 PI polyamide; Lane 4, FITC-dsDNA + excess unlabeled dsDNA + USF1 PI polyamide; Lane 5, FITC-dsDNA + mismatch PI polyamide; Lane 6, FITC-mutated dsDNA + USF1 PI polyamide.

Binding of the USF1 PI polyamide, designed to span the boundary of the USF1-binding E-box motif (CANNTG), to the target double-stranded DNA was evaluated by gel mobility shift assay. The USF1 PI polyamide bound to the FITC-labeled double-stranded DNA, as indicated by a shifted band (lane 3). Addition of excess unlabeled double-stranded DNA caused competitive binding to the USF1 PI polyamide between the unlabeled and FITC-labeled dsDNA, resulting in a marked reduction in complex formation and the predominant detection of the unshifted FITC-labeled dsDNA band (lane 4). In contrast, the mismatch PI polyamide did not cause a comparable mobility shift in the FITC-labeled target double-stranded DNA (lane 5). When the mutated dsDNA was incubated with the USF1 PI polyamide, the pronounced mobility shift observed with the target dsDNA was not detected (lane 6 vs. lane 3). These findings indicate that the USF1 PI polyamide preferentially binds to the intended target sequence (Figure 3). The competition experiment with excess unlabeled DNA, together with the lack of a comparable mobility shift with either the mismatch PI polyamide or mutated target DNA, further supported sequence-selective binding of the designed PI polyamide.

### 3.2. DNA Binding Assay for USF1 Polyamide

**Figure 4:**
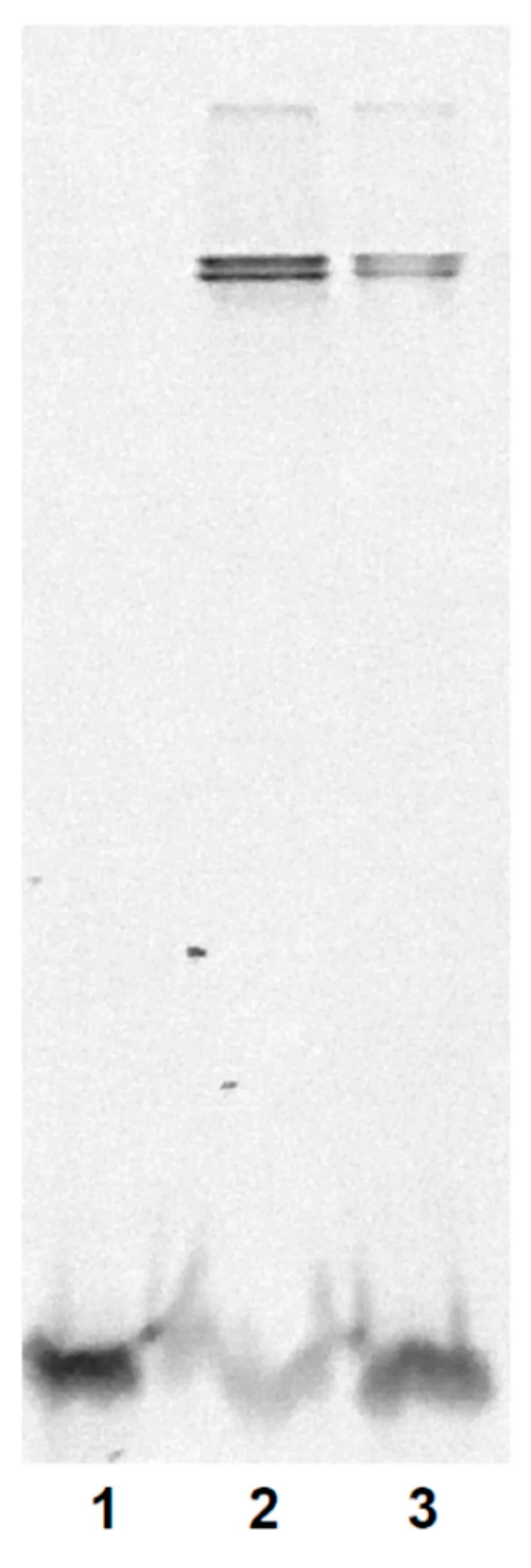
DNA binding assay for inhibition of USF1 binding by the pyrrole-imidazole (PI) polyamide. Sense and antisense oligonucleotides corresponding to the target sequence were synthesized, annealed to generate double-stranded DNA, and incubated with the USF1 PI polyamide for 1 h, followed by addition of recombinant human USF1 protein. Lane 1, FITC-dsDNA + USF1 PI polyamide; Lane 2, FITC-dsDNA + USF1; Lane 3, FITC-dsDNA + USF1 + USF1 PI polyamide.

A DNA binding assay was performed to determine whether the USF1 PI polyamide inhibited the interaction between USF1 and DNA. A shifted band was observed when the double-stranded DNA was incubated with recombinant USF1 protein (lane 2). This band was reduced in the presence of the USF1 PI polyamide (lane 3), indicating that the USF1 PI polyamide inhibited USF1 binding to the target DNA sequence (Figure 4). Thus, preincubation with the USF1 PI polyamide reduced formation of the recombinant USF1–DNA complex, consistent with interference with USF1 access to the target sequence.

### 3.3. Effects of USF1 PI Polyamide on TGF-β1 Expression in HRMCs Under High-Glucose Conditions

**Figure 5:**
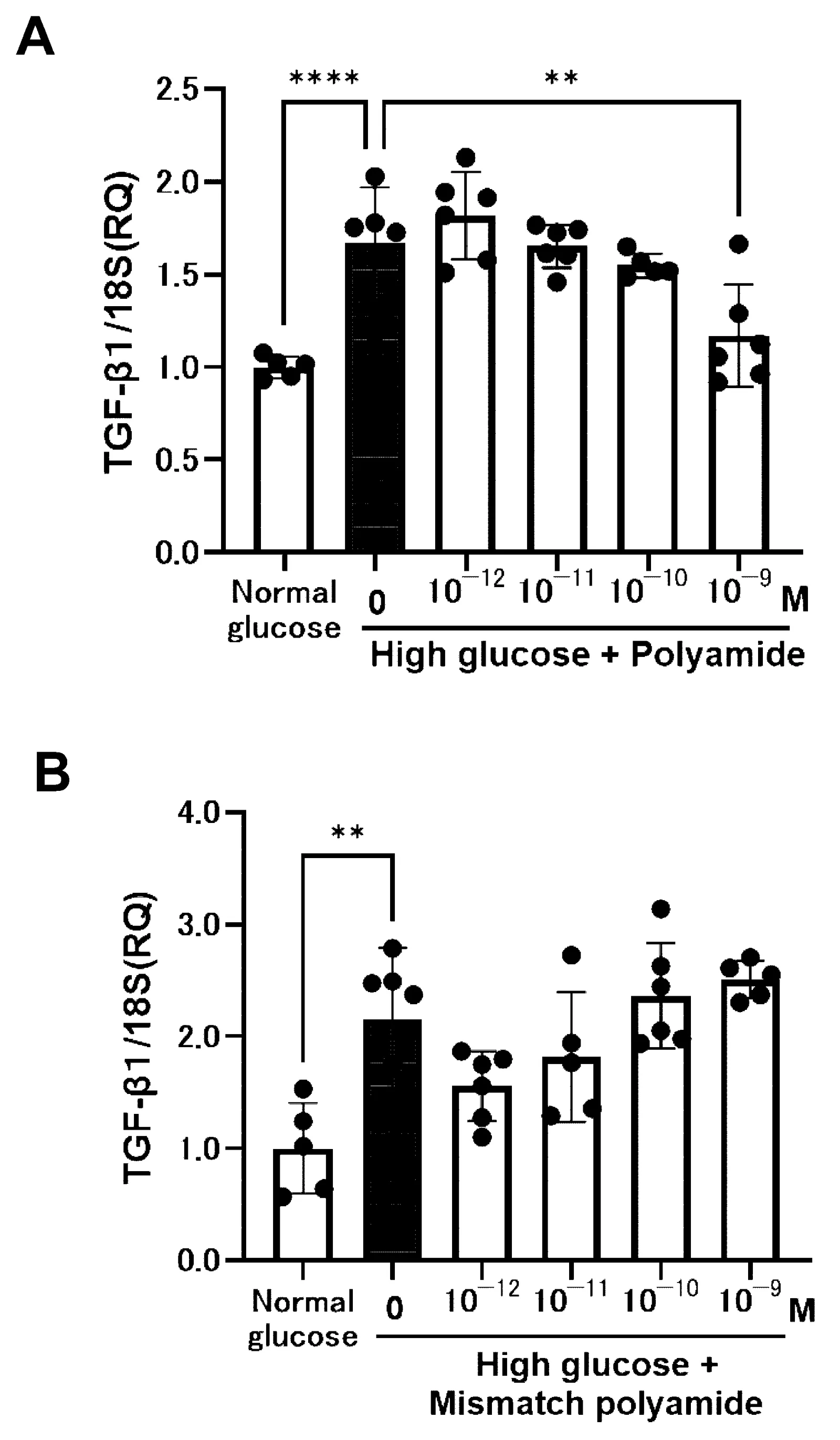

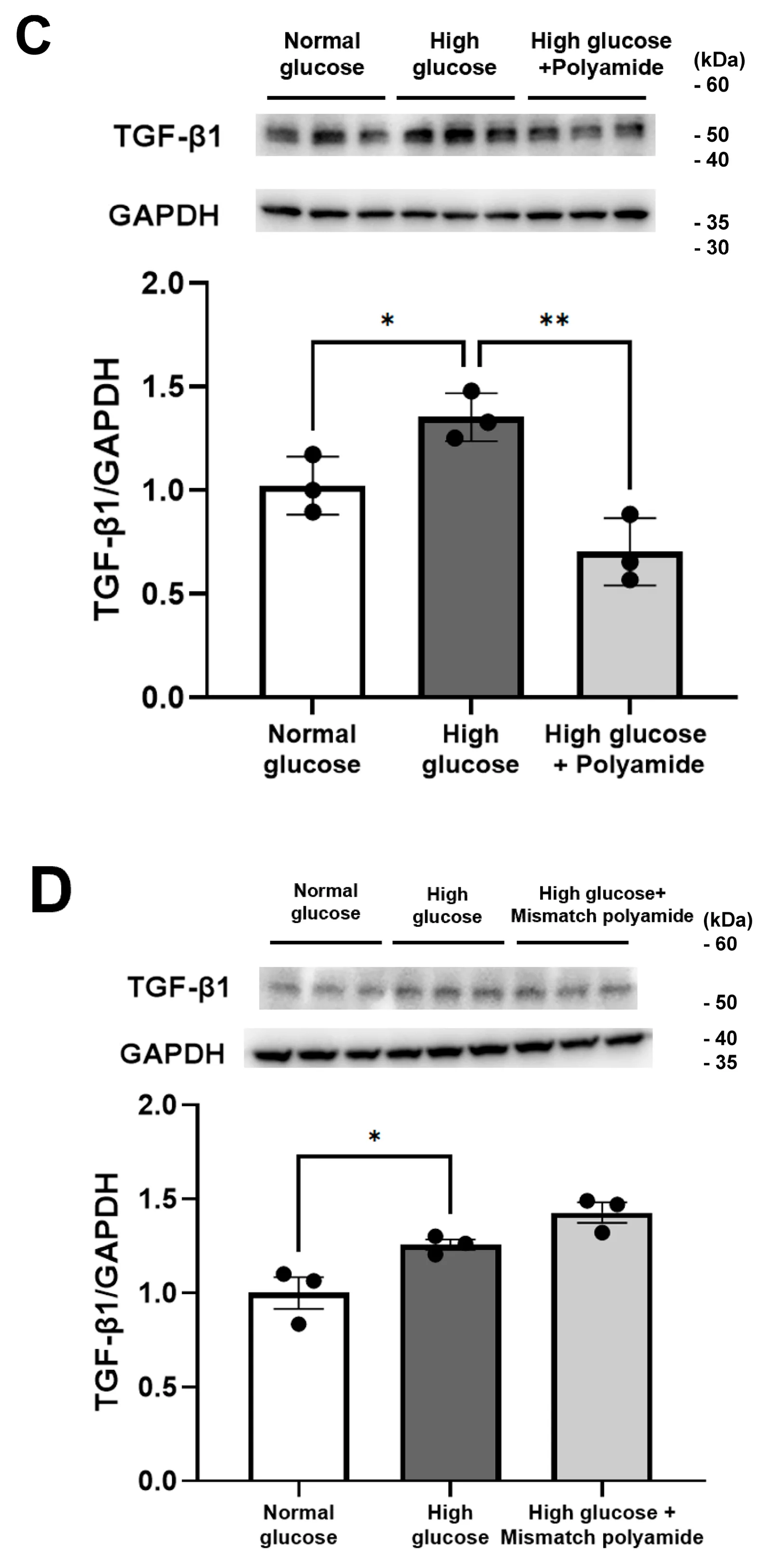
Effects of the USF1-targeting PI polyamide on TGF-β1 expression in high glucose-stimulated human renal mesangial cells (HRMCs). (**A**) Effects of the USF1 PI polyamide on TGF-β1 mRNA expression in HRMCs under high-glucose conditions, assessed by real-time PCR (*n* = 6). (**B**) Effects of the mismatch PI polyamide on TGF-β1 mRNA expression in HRMCs under high-glucose conditions, assessed by real-time PCR (*n* = 6). (**C**) Effects of the USF1 PI polyamide on TGF-β1 protein expression in HRMCs under high-glucose conditions, assessed by Western blotting (*n* = 3). (**D**) Effects of the mismatch PI polyamide on TGF-β1 protein expression in HRMCs under high-glucose conditions, assessed by Western blotting (*n* = 3). Data are presented as means ± SE. Statistical significance is shown for the indicated comparisons; * *p* < 0.05, ** *p* < 0.01, **** *p* < 0.0001.

High-glucose stimulation increased TGF-β1 mRNA expression in HRMCs. The USF1 PI polyamide significantly suppressed high-glucose-induced TGF-β1 mRNA expression at a concentration of 10^−9^ M (*p* < 0.01; Figure 5A). In contrast, the mismatch PI polyamide did not suppress the increase in TGF-β1 mRNA expression under high-glucose conditions (Figure 5B). In addition, 10^−9^ M USF1 PI polyamide significantly reduced TGF-β1 protein expression in HRMCs exposed to high glucose (*p* < 0.01; Figure 5C). In contrast, 10^−9^ M mismatch PI polyamide did not suppress TGF-β1 protein expression (Figure 5D). Across the concentrations tested, significant suppression of TGF-β1 mRNA was observed only at 10^−9^ M, whereas lower concentrations did not show significant suppression.

### 3.4. Effects of USF1 PI Polyamide on Osteopontin mRNA Expression in HRMCs Under High-Glucose Conditions

**Figure 6:**
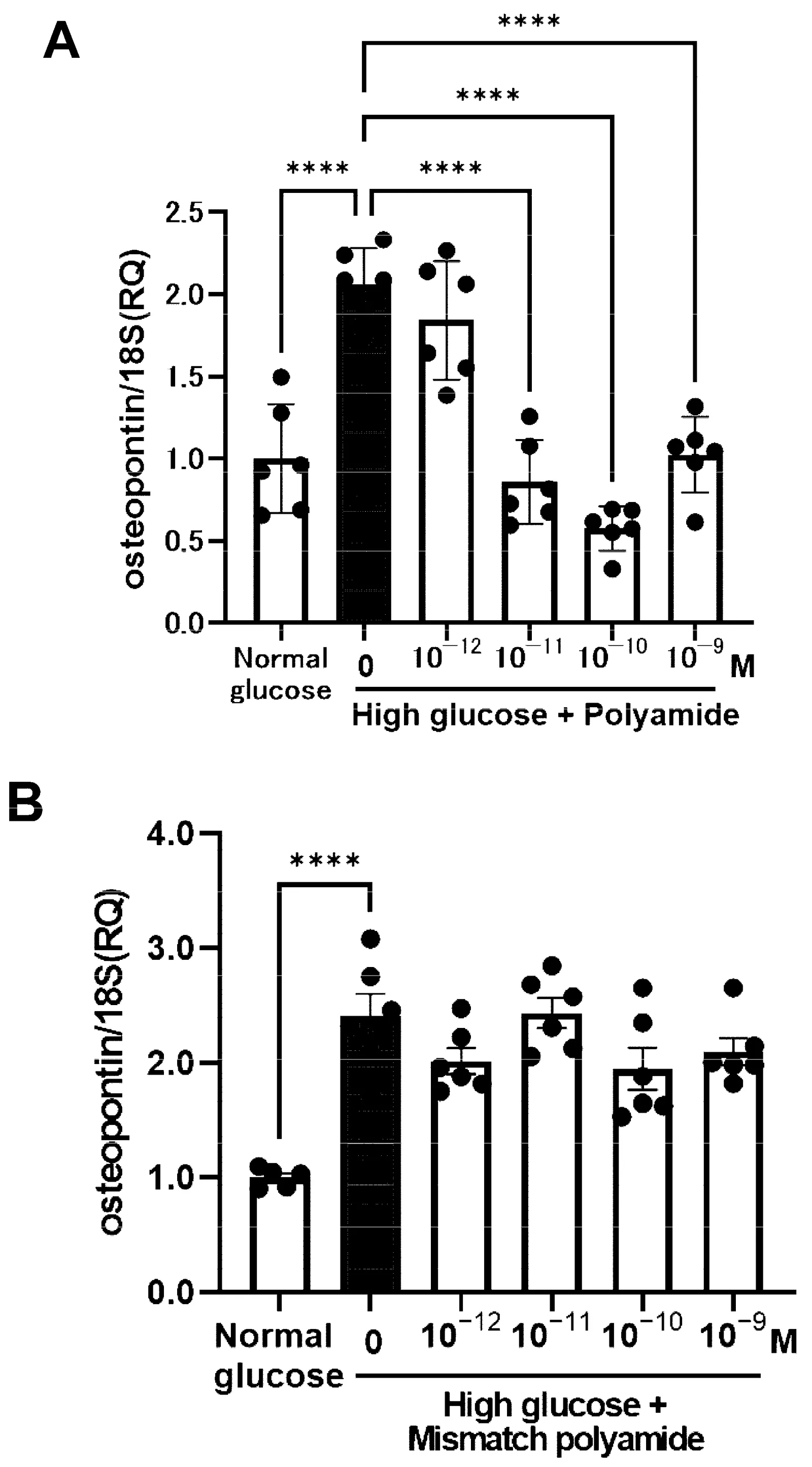
Effects of the USF1-targeting PI polyamide on osteopontin expression in high glucose-stimulated human renal mesangial cells (HRMCs). (**A**) Effects of the USF1 PI polyamide on osteopontin mRNA expression in HRMCs under high-glucose conditions, assessed by real-time PCR (*n* = 6). (**B**) Effects of the mismatch PI polyamide on osteopontin mRNA expression in HRMCs under high-glucose conditions, assessed by real-time PCR (*n* = 6). Data are presented as means ± SE. **** *p* < 0.0001.

High-glucose stimulation increased osteopontin mRNA expression in HRMCs. The USF1 PI polyamide significantly suppressed high-glucose-induced osteopontin mRNA expression at concentrations of 10^−11^ to 10^−9^ M (*p* < 0.0001; Figure 6A). In contrast, the mismatch PI polyamide did not suppress the increase in osteopontin mRNA expression under high-glucose conditions (Figure 6B). Notably, significant suppression of osteopontin mRNA was observed over a broader concentration range than that observed for TGF-β1 mRNA, whereas no comparable reduction was observed with the mismatch PI polyamide.

## 4. Discussion

Diabetic kidney disease is characterized by mesangial matrix expansion, glomerulosclerosis, and progressive fibrosis. Although numerous factors are involved in these pathological changes, TGF-β1 is known to induce extracellular matrix accumulation, glomerular hypertrophy via mesangial proliferation, and both epithelial-to-mesenchymal transition (EMT) and endothelial-to-mesenchymal transition (EndMT)—leading to tubulointerstitial fibrosis and glomerular endothelial cell injury—thereby playing a central role in the pathogenesis of diabetic nephropathy [3,15]. In the diabetic milieu, high glucose activates multiple intracellular pathways and transcription factors that enhance TGF-β1 expression [8]. In particular, USF1 has been shown to translocate to the nucleus under high-glucose conditions and to activate TGF-β1 transcription through the glucose-response element/E-box motif in the promoter region [10]. These observations support the biological importance of the USF1–TGF-β1 axis in diabetic kidney disease.

Previous experimental studies have suggested that this pathway may be therapeutically targetable. Okamura et al. reported that a PI polyamide designed to prevent USF1 binding to the rat TGF-β1 promoter suppressed TGF-β1 expression and ameliorated glomerular and tubular injury in diabetic rats [7]. However, the activity of such a strategy against the human TGF-β1 promoter has not been sufficiently examined. In the present study, we therefore designed and synthesized a PI polyamide targeting the human USF1-binding site in the TGF-β1 promoter and evaluated its effects in human renal mesangial cells. Because mesangial cells are an important source of autocrine TGF-β1 under high-glucose conditions [12], this cell system was considered appropriate for examining the relevance of this transcriptional regulatory mechanism in humans.

The present study provides several findings. First, the designed PI polyamide bound specifically to the target double-stranded DNA sequence, as shown by a gel mobility shift assay. Second, the USF1 PI polyamide inhibited the interaction between recombinant USF1 protein and the target DNA sequence in vitro. Third, in human renal mesangial cells exposed to high glucose, the USF1-targeting PI polyamide suppressed TGF-β1 mRNA expression and reduced TGF-β1 protein abundance, whereas the mismatch PI polyamide did not show such an effect. Together, these results support the interpretation that the observed suppression of TGF-β1 expression was sequence-dependent and consistent with inhibition of USF1 binding to the human TGF-β1 promoter [10].

In addition to TGF-β1, we examined osteopontin expression as a marker related to mesangial injury. Osteopontin is a stress-responsive matrix-associated cytokine that has been linked to albuminuria, glomerulosclerosis, and mesangial injury in diabetic kidney disease [13]. In the present study, high glucose increased osteopontin mRNA expression in human renal mesangial cells, and this increase was suppressed by the USF1-targeting PI polyamide. Because USF1 has also been implicated in transcriptional regulation through E-box motifs [10], this finding is consistent with the possibility that inhibition of USF1-related transcriptional activity may attenuate not only TGF-β1 upregulation but also broader profibrotic or phenotypic responses in mesangial cells. The finding that osteopontin expression was suppressed across a broader concentration range than TGF-β1 might suggest that osteopontin downregulation is not driven solely by TGF-β1 inhibition but may also involve TGF-β1-independent mechanisms, including direct interaction of the PI polyamide with compatible sequence motifs within the osteopontin regulatory region. Even in the absence of an exact sequence match, hairpin PI polyamides recognize base pairs via canonical pairing rules (Py/Im for C–G, Im/Py for G–C, Py/Py for T–A/A–T, and γ-turn for T–A/A–T), permitting engagement with closely related promoter motifs. Additionally, polyamide-induced allosteric modulation of DNA architecture [16] may alter local promoter accessibility, potentially accounting for the concentration-dependent divergence between the two genes. However, direct binding to osteopontin regulatory sequences was not examined in this study, and meticulous evaluation to define the optimal effective concentration range will be essential in future studies.

Several limitations should be acknowledged. First, this was an in vitro study using a single human mesangial cell system, and the present data do not establish efficacy in vivo. Second, although high glucose at 25 mM has previously been reported to induce transcriptional responses in mesangial cells [10,17], an osmotic control was not included in the present experiments. Previous studies using mesangial cells have shown that mannitol controls did not reproduce the high-glucose-induced changes in TGF-β1 or osteopontin expression [17,18,19], suggesting that osmotic stress alone is unlikely to fully account for the observed responses. Nevertheless, because osmotic control was not included in our own HRMC experiments, the contribution of osmotic stress cannot be completely excluded.

Third, the PI polyamide was designed to recognize a short sequence within the USF1-binding region and may therefore bind to genomic regions other than the intended target, raising the possibility of off-target effects. Although the mutated DNA did not show the pronounced mobility shift observed with the target sequence, binding specificity was evaluated using a limited set of oligonucleotide controls, and potential interactions with other genomic sequences cannot be excluded. This point is particularly relevant because the suppression of osteopontin expression suggests that genes other than TGF-β1 may also be influenced. In addition, USF1 is not kidney-specific, and the potential effects of this approach in other cell types or organs remain unknown. The specificity of PI polyamides is supported by previous microarray profiling by Matsuda et al., which revealed that non-target transcripts affected by PI polyamide administration were minimal and predominantly associated with the target pathway. Crucially, PI polyamides preferentially suppress pathologically upregulated transcriptional activity without disrupting basal gene expression in quiescent states [6]. In the context of diabetic nephropathy, where TGF-β1 and osteopontin are intertwined, the observed simultaneous downregulation indicates that the USF1 PI polyamide may preferentially affect the hyperglycemia-induced pathological elevation of osteopontin. To further confirm these findings and map global selectivity, future studies utilizing genome-wide transcriptome profiling, such as RNA-seq, will be essential.

Despite these limitations, the present findings are meaningful in that they extend previous rodent-based observations [7] to the human TGF-β1 promoter and demonstrate proof-of-concept in human renal mesangial cells. PI polyamides have characteristics that make them attractive as transcriptional modulators, including sequence specificity, nuclease resistance, and cellular permeability [4,5]. Moreover, PI polyamides designed to interfere with transcription factor binding are thought to suppress disease-associated promoter activation without directly silencing basal gene expression [6]. On this basis, targeting the human USF1–TGF-β1 axis may represent a promising strategy for further translational investigation in diabetic kidney disease.

## 5. Conclusions

We designed and synthesized a PI polyamide targeting the human USF1-binding site in the TGF-β1 promoter. This PI polyamide inhibited USF1 binding to target DNA in vitro and attenuated high glucose-induced TGF-β1 and osteopontin expression in human renal mesangial cells. These findings provide proof-of-concept for targeting the human USF1–TGF-β1 axis and support further in vivo and translational studies.

## Data Availability

The original contributions presented in this study are included in the article/supplementary material. Further inquiries can be directed to the corresponding authors.

## Supplementary Materials

The following supporting information can be downloaded at: https://www.mdpi.com/article/10.3390/cells15191818/s1, Table S1: qPCR primers and TaqMan probes used in this study.
