# Supplementary material for: A Pyrrole-Imidazole Polyamide Targeting Human USF1 Binding Suppresses High Glucose-Induced TGF-β1 and Osteopontin Expression in Human Renal Mesangial Cells
Source: Cells. 2026 Oct 6;15(19):1818. doi: 10.3390/cells15191818 (PMC13664332; doi:10.3390/cells15191818)
Supplement: Supplementary file 1 [file cells-15-01818-s001.zip › cells-4428344-supplementary.pdf]

cells

MDPI

Article

A Pyrrole-Imidazole Polyamide Targeting Human USF1 Binding Suppresses High Glucose-Induced TGF-β1 and Osteopontin Expression in Human Renal Mesangial Cells

Yurie Akiya [1], Hiroki Kobayashi [1,*], Akiko Tsunemi [1], Noboru Fukuda [2] and Masanori Abe [1]

[1] Division of Nephrology, Hypertension, and Endocrinology, Department of Internal Medicine, Nihon University School of Medicine. 30-1 Oyaguchi Kami-chou, Itabashi-ku, Tokyo 173-8610, Japan; akiya.yurie@nihon-u.ac.jp (Y.A.); tsunemi.akiko@nihon-u.ac.jp (A.T.); abe.masanori@nihon-u.ac.jp (M.A.)

[2] Division of Nephrology and Endocrinology, Ukima Chuo Hospital, Tokyo, Japan; fukuda.noboru57@gmail.com

* Correspondence: kobayashi.hiroki@nihon-u.ac.jp; Tel.: +81-3-3972-8111; Fax: +81-3-3972-8311

Supplementary Materials

Supplementary Table S1. List of primers and TaqMan probes used for qPCR.

18S	Probe	[6FAM]CTTGCGTTGATTAAGTCCCTGCCCTTTGT[TAM]
	Sense	5′-TCCCAGTAAGTGCGGGTCA-3′
	Anti-sense	5′-CCATCCAATCGGTAGTAGCGA-3′
TGF-β1	Probe	[6FAM]CCCTACATTTGGAGCCTGGACACGCAGTA[TAM]
	Sense	5′-CTCGGCTGGAAGTGGATCCA-3′
	Anti-sense	5′-TGTACAGGGCCAGGACCTTG-3′
osteopontin	Sense	5′-AATACCCAGATGCTGTGGCC-3′
	Anti-sense	5′-CTGGCTGTCCACATGGTCAT-3′

Abbreviations: TGF-β1: transforming growth factor-β1.
